# Needle‐ and Canopy‐Level Genetic Variation in Scots Pine (*Pinus sylvestris* L.) Revealed by Hyperspectral Phenotyping Across Sites and Seasons

**DOI:** 10.1111/eva.70176

**Published:** 2025-11-12

**Authors:** Daniel Provazník, Jan Stejskal, Zuzana Lhotáková, Jaroslav Čepl, Eva Neuwirthová, Adenan Yandra Nofrizal, Jiří Korecký, Lucie Červená, Lucie Kupková, Jaroslav Klápště, Jon Kehlet Hansen, Salvador A. Gezan, Petya Campbell, Milan Lstibůrek, Jana Albrechtová

**Affiliations:** ^1^ Faculty of Forestry and Wood Sciences Czech University of Life Sciences Prague Prague Czech Republic; ^2^ Department of Geosciences and Natural Resources Management University of Copenhagen Copenhagen Denmark; ^3^ Department of Experimental Plant Biology, Faculty of Science Charles University Prague Czech Republic; ^4^ Department of Applied Geoinformatics and Cartography, Faculty of Science Charles University Prague Czech Republic; ^5^ Tree Breeding Australia Mount Gambier Australia; ^6^ VSN International Hemel Hempstead UK; ^7^ Department of Geography and Environmental Sciences University of Maryland, Baltimore County Baltimore Maryland USA; ^8^ Biospheric Sciences Laboratory NASA Goddard Space Flight Center Greenbelt Maryland USA

**Keywords:** clonal seed orchards, genetic correlations, heritability, needle functional traits, spectral reflectance, UAV

## Abstract

As an essential species across European forests, Scots pine ( *Pinus sylvestris* L.) plays a vital ecological and economic role, yet its physiological variability underlying its adaptive potential remains underexplored. Understanding this intraspecific variability is crucial for uncovering the genetic basis of adaptation. Traditional genetic evaluations require large sample sizes and are time‐consuming, whereas hyperspectral sensing/imaging enables rapid, nondestructive assessment of physiological traits across many individuals, facilitating more efficient exploration of adaptive variation. We assessed needle functional traits (NFTs) linked to foliar structure, water content, and pigment composition in clonal seed orchards over two seasons, integrating hyperspectral measurements at needle and canopy levels with genotyping using a new 50 K single‐nucleotide polymorphism (SNP) array. Linear mixed models revealed substantial genetic variation, with the carotenoid‐to‐total‐chlorophyll ratio showing the highest heritability (0.29) among pigment traits, and structural/water‐related traits reaching heritability values up to 0.38. Significant genetic correlations were observed between stress‐related traits (pigment content, equivalent water thickness) and reflectance, suggesting that spectral traits could serve as proxies for indirect selection of adaptive traits or in breeding programs. Low genotype‐by‐environment interaction and stable clonal performance across years further underscore the reliability of these traits for identifying resilient genotypes. Overall, our findings highlight hyperspectral phenotyping and NFTs as promising tools for accelerating climate‐adaptive breeding in Scots pine.

## 1 Introduction

Scots pine ( *Pinus sylvestris* L.) forests play a key ecological and economic role, which is likely to increase with ongoing climate change. Scots pine is a pioneer tree species (Linder et al. 1997) characterized by sizable genetic variability within populations but also by phenotypic plasticity, which enables the species to cover a large natural range across Eurasia (Brichta et al. 2023). Despite the recent decline of conifers in Europe, Scots pine remains an essential tree species for European forests (Buras and Menzel 2019; Ols and Bontemps 2021). Clonal seed orchards of Scots pine offer an experimental framework, allowing for the examination of genetic variability and adaptive responses by measuring phenotypes across replicated genotypes (Stejskal et al. 2022).

In tree populations where pedigree or family structure is unavailable, genomic relationship matrices (GRMs) derived from single‐nucleotide polymorphism (SNP) data enable the estimation of additive genetic variance, genetic correlations, and other quantitative genetic parameters (Yang et al. 2010). These matrices quantify realized genetic relatedness among individuals using genome‐wide marker information and provide a powerful alternative to traditional pedigree‐based approaches, especially for outcrossing and long‐lived organisms such as forest trees (De Los Campos et al. 2015; Grattapaglia and Resende 2011). GRMs facilitate the application of genomic best linear unbiased prediction (GBLUP) and mixed‐model frameworks, allowing for robust partitioning of phenotypic variance into genetic and environmental components even in the absence of known familial relationships (VanRaden 2008).

GRMs can also be integrated with phenotypic information derived from emerging high‐throughput phenotyping methods. One such approach is hyperspectral remote sensing, which provides a powerful way to capture fine‐scale variation in vegetation traits and ecosystem dynamics with unmatched detail (Zhang et al. 2021). Leaf reflectance spectra are effective tools in studies of trait differentiation. Different wavelengths can serve as proxies for specific chemical or physiological traits, as well as indicators of plant health (Corbin et al. 2024). For example, bands in the visible region (VIS) of a plant's spectral reflectance signal are associated with photosynthetic pigment content (Gitelson and Solovchenko 2018; Sims and Gamon 2002), in the near‐infrared (NIR) region with structural compounds (Buschmann et al. 2012), and in the short‐wave infrared (SWIR) region with water content (Granlund et al. 2018). Another important spectral domain is the transition between the VIS and NIR regions, commonly referred to as the red edge. The red‐edge region associated with chlorophyll concentration (Curran et al. 1995) can be utilized for early stress detection caused by various factors such as drought (Bloom et al. 2025), girdling (Eitel et al. 2011), or bark beetle attacks (Abdullah et al. 2019; Ahern 1988). Other traits, such as leaf phenology, can be related to specific bands across these regions (Campbell et al. 2019; Neuwirthová et al. 2021). Moreover, NIR‐related structural information can harbor information on population origin (Stejskal et al. 2023).

Several studies have investigated how spectral and physiological traits in Scots pine respond to environmental and genetic factors, such as altitude, age, and genotype. For example, Dengel et al. (2013) found that the spectral reflectance of pine needles can indicate chlorophyll degradation in Scots pines growing at higher elevations. Similarly, needle reflectance patterns indicate adaptive variation among Scots pine provenances on the intrapopulation scale (Čepl et al. 2018; Danusevicius et al. 2014).

This capability of hyperspectral methods to link physiological and structural traits with genetic factors provides a foundation for estimating heritability and genetic correlations among traits. Traditional methods of genetic evaluation require large sample sizes—typically hundreds of individuals—which can be time‐consuming and costly. Optical methods, particularly hyperspectral imaging, offer a promising solution by enabling the efficient assessment of large samples (Bian et al. 2022). Measuring needle optical properties in the laboratory using a contact probe spectroradiometer offers key advantages, including a stable light source, fixed measurement geometry, and high spectral resolution across a broad range (350–2500 nm). However, data collection with contact probes can still be too time‐consuming for the large sample size required for genetic evaluation. Hyperspectral cameras can overcome this hurdle when mounted on unmanned aerial vehicles (UAVs), capturing spectral image data with high spatial and spectral resolution over large areas. This feature is particularly advantageous in progeny or clonal trials, obtaining a considerably larger sample size crucial for genetic evaluation (Grubinger et al. 2023). Unlike spectroradiometers, hyperspectral image data require segmentation, a task complicated in forest environments by shadows (Kopačková‐Strnadová et al. 2021) and understory vegetation. The camera's spatial resolution is another critical parameter affecting data quality (Feduck et al. 2018). In our study, we utilized both approaches, using data from a lab‐based spectroradiometer and hyperspectral image data obtained with a camera mounted on a UAV.

Despite these complexities, spectral traits are used for indirect selection in crop breeding (Al‐Ashkar et al. 2021; El‐Hendawy et al. 2020). High‐resolution spectral data, advanced machine learning, and genetic analysis have also been integrated into indirect selection in tree breeding for forestry. Researchers have applied a range of approaches from biochemical trait prediction (Song et al. 2024), ecotypic and physiological trait characterization (Hejtmánek et al. 2022), population differentiation and provenance identification (Stejskal et al. 2023), to seasonal and climatic adaptation assessment (Grubinger et al. 2023). The basis of indirect selection within breeding programs lies in leveraging genetic correlations between spectra and any of the considered production traits or adaptive traits, such as needle functional traits (NFTs). For instance, one of the NFTs, the equivalent water thickness (EWT), can be used to assess forest canopy health and water status (Li et al. 2021; Wocher et al. 2018), particularly in response to various threats related to climate change—wildfires, intense heatwaves, and consecutive insect outbreaks.

Remote sensing methods have significantly advanced ecological studies over the past decades, yet their use in tree physiology and forest genetics is relatively recent (Čepl et al. 2018; D'Odorico et al. 2023; Grubinger et al. 2023; Wong et al. 2019). While earlier research has primarily focused on large‐scale ecosystem analyses, finer‐scale genetic and physiological variations within species—such as Scots pine—and the interactions between genotypes and dynamic environments across space and time have often been overlooked. This gap is critical, as neglecting genotype‐by‐environment interactions (G × E) can lead to substantial production losses in operational forestry (Zobel and Talbert 1984). G × E caused by rank changes or scale effects has been reported in many tree species (White et al. 2007). Scots pine's type B genetic correlations (a measure of G × E interaction) have been reported for traits like height (Hejtmánek et al. 2023) and vitality (Calleja‐Rodriguez et al. 2019). However, type B genetic correlations for hyperspectral reflectance traits in Scots pine have not yet been thoroughly investigated. Similarly, the genetic correlations between reflectance and NFTs—critical for enabling indirect selection—have received limited attention. Addressing this aspect requires a multidisciplinary approach that bridges remote sensing, quantitative genetics, genomics, and plant physiology (Cavender‐Bares et al. 2020; Čepl et al. 2018).

In this study, conducted in replicated clonal seed orchards of Scots pine, we aimed to test specific hypotheses regarding genetic variation in NFTs and needle‐ and canopy‐level spectral reflectance using a newly developed 50 K SNP genotyping array (Kastally et al. 2022). To capture both genetic and physiological variation among clones, we integrated SNP‐based genotyping with hyperspectral phenotyping, enabling high‐resolution, genome‐informed modeling of heritability and genetic correlations. This combination improves the power and accuracy of quantitative genetic analyses by leveraging marker‐derived GRMs alongside rich phenomic data (Grattapaglia et al. 2018; Resende et al. 2012). Such integrative approaches are especially valuable in forestry, where indirect selection for adaptive traits—such as drought tolerance, pigment composition, or water status—can benefit from linking dense genomic information with nondestructive, high‐throughput trait assessments. Ultimately, this framework facilitates genomic‐informed breeding strategies aimed at enhancing the resilience of forest trees to climate change (Grattapaglia et al. 2018; Holliday et al. 2017; Neale and Kremer 2011). We hypothesized that hyperspectral data would exhibit significant genetic variation and reveal genetic correlations with NFTs at needle and canopy levels. Additionally, we investigated the extent of year‐to‐year and site‐by‐site (type B) genetic correlation in reflectance, as high correlation values indicate stable trait expression across years or environments (low GxE). Lastly, we investigated the extent of genetic correlations between NFTs and spectral reflectance, which indicates the potential for indirect selection based on needle optical properties.

## 2 Materials and Methods

### 2.1 Plant Material

Two first‐generation clonal seed orchards of Scots pine were selected for this study. The seed orchards are referred to by their neighboring towns, Plasy and Nepomuk. Both seed orchards were established by grafting scions from plus trees (superior individual trees selected from a population based on their growth and vitality). Both sites are located in the western part of Czechia at coordinates 49°54′31.543″ N, 13°26′33.860″ E (Plasy) and 49°28′53.747″ N, 13°31′38.760″ E (Nepomuk), approximately 50 km apart. The soil properties slightly differ between the orchards, particularly in magnesium, phosphorus, and nitrogen content and in carbon‐to‐nitrogen ratio (Table S1). Plasy clonal seed orchard (6.48 ha) was established in 1980 with 87 grafted clones and 1165 ramets (technical term for a clonal replication). Nepomuk clonal seed orchard (2.24 ha) was established in 1975 with 45 grafted clones and 410 ramets. There was an overlap of 24 genotyped clones across both seed orchards. The design with all ramets of all genotyped clones is visualized in Figure S1 for Plasy and Figure S2 for Nepomuk.

For the laboratory spectral and NFT measurements, we collected samples from 148 ramets of 29 clones at Plasy in 2021, and 200 ramets of 31 clones at Nepomuk in 2021. In 2021, we sampled up to 7 ramets of each clone if they were available in the given seed orchard. Some clones suffered mortality or were not planted in such quantity; in an extreme case at Plasy, only 1 ramet of a clone was available. We did not include any leaf‐level measurements from 2020 because the dataset lacked sufficient representation of replicated clones needed for reliable genetic analysis.

**FIGURE 1:**
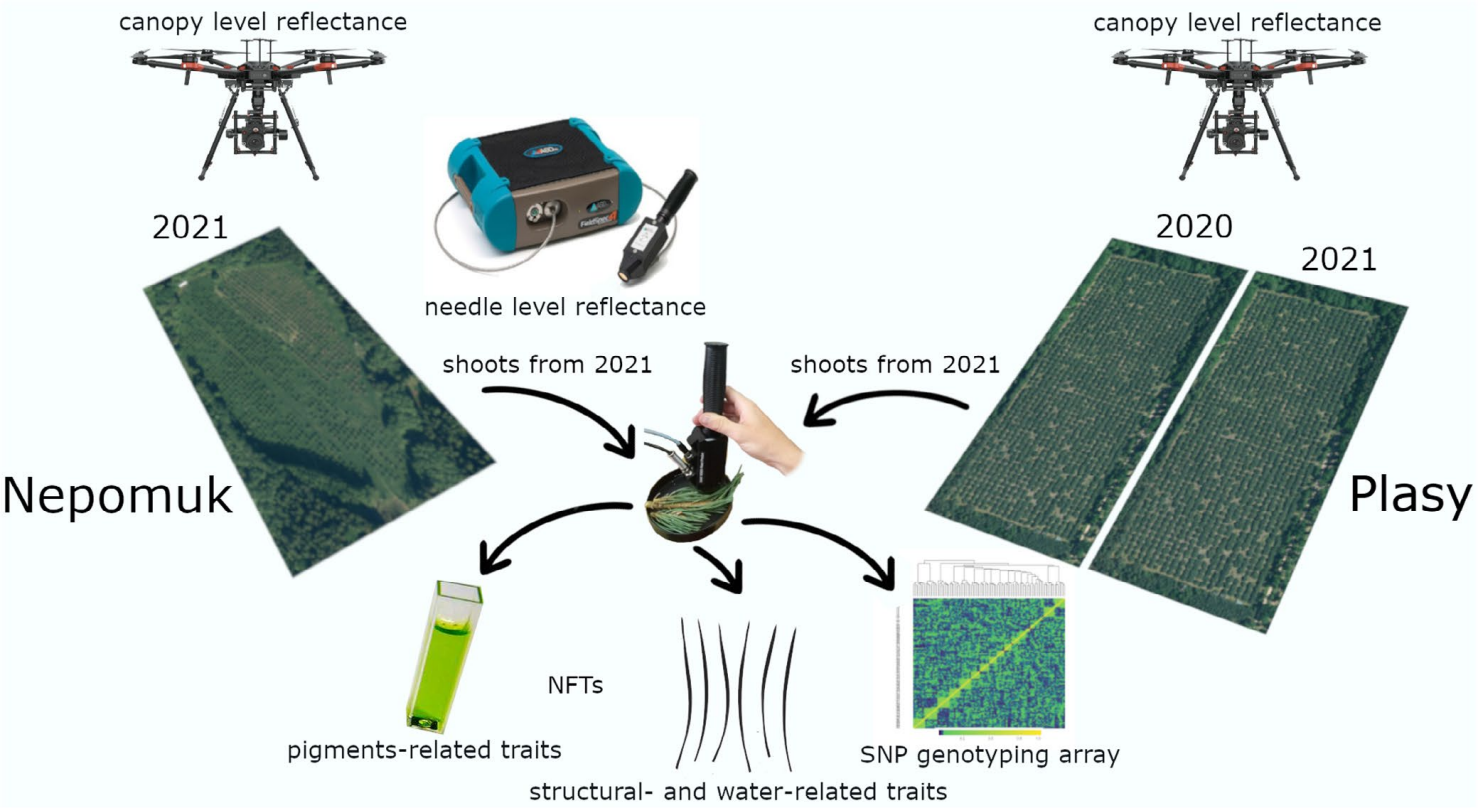
Graphical abstract illustrating the workflow of individual measurements and analyses conducted for specific years and sites.

We collected fully sun‐exposed branches from the mid‐to‐upper crown's southern side using telescopic pole‐scissors. Samples were collected between 10:00 am and 4:00 pm during a single day at each site. Branches were at least 100 cm long to minimize desiccation, with stumps wrapped in moistened towels. At the end of the day, they were transported to the laboratory, stored at ~4°C, and processed the following morning. Collection times were recorded, and spectral measurements were completed within 24 h of cutting, maintaining sample order. The sampling protocol was tuned according to (Čepl et al. 2018, 2016), and according to the results of Richardson and Berlyn (2002) showing stable chlorophyll fluorescence and reflectance values in evergreen conifers 48 h after branch cutting. We present a workflow illustration (Figure 1) outlining this study's methods.

### 2.2 Needle‐Level Reflectance

To evaluate reflectance at the needle level, we used a FieldSpec 4 Wide‐Res portable spectroradiometer (Malvern Panalytical Ltd., United Kingdom) equipped with a detachable contact probe (Malvern Panalytical Ltd., United Kingdom). The contact probe has an internal light source, allowing it to measure needle‐level reflectance from 350 to 2500 nm. The spectroradiometer contains three sensors that cover the spectral range 350–1000 nm (resolution 3 nm), 1000–1800 nm (resolution 8 nm), and 1800–2500 nm (resolution 8 nm). For calibration, a white Spectralon panel (SphereOptics, Herrsching am Ammersee, Germany) with approximately 99% reflectance in each wavelength was used every 15 min. Shoots (collected also for the NFT measurements) of sampled branches with needles attached were placed on a plate coated with a spectrally black coating (NEXTEL Velvet‐Coating 811–21) with low reflectance across the measured spectral region. Needles from the top of the branch were arranged together to cover the entire top of the contact probe, consistent with methods used in previous studies (Čepl et al. 2018; Hejtmánek et al. 2022). The scan average on the spectroradiometer was set to 25. Reflectance was calculated as the ratio between the radiance of the white panel and the radiance measured from the sample. The median reflectance was determined from five sample measurements after branch rotation and needle rearrangement. The spectral reflectance and NFTs were obtained within 24 h after the collection.

### 2.3 Canopy‐Level Reflectance

Hyperspectral image data were acquired using the Headwall Nano‐Hyperspec manufactured by Headwall Photonics Inc. (Fitchburg, Massachusetts, USA) camera mounted on the DJI Matrice 600 Pro UAV. The spectral range of the camera is from 400 to 1000 nm and is covered by 269 bands. All the flights were conducted around midday to have the sun as high as possible. At Plasy in September 2021, spectra were acquired under a cloudy sky, which should not affect the results significantly. Although the signal‐to‐noise ratio (SNR) in the data acquired under a cloudy sky is lower, diffuse illumination can enhance the spectral features of vegetation (Arroyo‐Mora et al. 2021; Shoshany et al. 2019). The flight altitude was 68.9 m above the ground to get a ground sampling distance of 3 cm. The side overlap of flight lines was set to 40% to ensure that every tree would be in at least one flight line as a whole tree. Radiometric and basic geometric corrections were performed in Headwall SpectralView—Hyperspec v3.1.0 software. The radiometric corrections were performed using the portable fabric target with known reflectance placed in the scanned area (Group 8 Technology Inc., Provo, UT, USA). The geometric corrections were performed using GNSS and IMU logs recorded by the UAV. The orthorectification was performed using the global digital elevation model provided by Headwall software (pixel size 0.0083°), as a digital surface model with trees is unavailable. The seed orchards' terrain is flat, and the orthophoto mosaic was not generated. All parameters for data acquisitions are summarized in Table 1.

**TABLE 1:** Summary of flight conditions when canopy‐level data were acquired.

Seed orchard/year	Plasy 2020	Plasy 2021	Nepomuk 2021
Date	05/08/2020	17/09/2021	25/08/2021
Local time	11:59–14:30	12:33–13:18	13:09–13:39
Weather conditions	Clear sky	Overcast	Clear sky
Exposure	3.996 ms	5.995 ms	5.995 ms
Number of flights	4	2	2
Number of flight lines	16	16	11

The initial data processing involved classifying the green canopy needles, branches, undergrowth vegetation, soil, and shadows utilizing hyperspectral image data to obtain spectral reflectance at the canopy level. Object‐Based Image Analysis (OBIA) was conducted using the ENVI software (version 5.5) and a Feature Extraction toolbox. Segmentation (Jin 2012) was performed using the scale level 30 and merge level 85 based on all bands and spatial properties of the data. The scale parameter determines the size of the segments, while the merge level parameter controls the extent to which neighboring segments are merged.

Fifty samples were selected for each of the five classes based on visual interpretation of the image for training and validation of classifications. Then, they were divided randomly into a 3:2 ratio, i.e., 30 samples for training, 20 samples for validation per class. Spectral features (mean, standard deviation, minimum, and maximum), texture features (range, mean, variance, and entropy), and spatial features (area, length, compactness, convexity, and solidity) were considered for OBIA classification using the Support Vector Machine (SVM) algorithm, which employs a kernel‐type radial basis function.

The results demonstrated satisfactory classification accuracy with an overall accuracy of more than 85% for both Plasy and Nepomuk. For the class of interest, canopy green leaves, the results were even better (producer's accuracy of 94% and user's accuracy of 90% for the Plasy area, 92% and 88% for the Nepomuk area, respectively). The zonal statistic multiband toolbox from the Dymaxion Labs plugin in QGIS (QGIS Development Team 2024) was used to extract the mean reflectance for every tree (class green canopy needles only) from 269 bands of hyperspectral imagery data.

Due to a systematic error of fluctuation of reflectance values in every second wavelength, each pair of neighboring wavelengths was averaged, resulting in smooth reflectance data. Additionally, the first 13 bands at the beginning and the last 36 bands at the end of the spectral range were dropped before analyzing to eliminate spectral noise, leaving 220 bands for the analysis (429–919 nm).

Hyperspectral image data were acquired for whole seed orchards, capturing all present trees. Only the data from ramets of the genotyped clones were analyzed (984 ramets of 67 clones at Plasy, and 261 ramets of 25 clones at Nepomuk). The spatial visualization is presented in Figure S1 for Plasy and Figure S2 for Nepomuk seed orchard.

### 2.4 Measurement of Needle Functional Traits

Needle samples for NFT assessment were collected from the branches used for needle‐level reflectance measurements. Branches with current‐year shoots were sampled. Two needle samples (3 current‐year needles per sample) were collected by detaching from each shoot: one for pigment content and the second for water content and leaf mass per area (LMA) assessment. The first needle sample was used for the biochemical evaluation of photosynthetic pigments (chlorophyll a (chl a) and chlorophyll b (chl b); total chlorophylls (chl T); total carotenoids (car)). Needles were cut into 2–3 mm segments, sealed in plastic vials, and stored at −20°C before biochemical analyses. The pigments were extracted in the dark at 4°C for 7 days using N‐dimethylformamide (Porra et al. 1989). The extract absorbance in 480, 647, 664, and 750 nm was determined spectrophotometrically using a Spectrophotometer (Evolution 201, Thermo Fisher Scientific, Waltham, MA, USA), and pigment concentrations were calculated according to Wellburn (Wellburn 1994) and related to dry weight (mg of pigment per g of dry mass). A ratio of carotenoids to total chlorophylls (car:chl T) was additionally calculated from the obtained values.

The second needle sample was used to assess water content and LMA. The needles were sampled in the laboratory the subsequent day after collection (see Section 2.1). Needles were weighed fresh, scanned (EPSON Perfection V600 Photo scanner with upper lamp, resolution 800 dpi, grayscale), and dried (60°C to constant weight). Needle projection area and needle length were determined on binary images in ImageJ (National Institutes of Health, Bethesda, Maryland, USA). LMA was calculated as the dry weight of the needle sample related to the needle sample projection area (g/cm^2^). LMA was used as one of the needle traits and for recalculating other needle traits (pigment and water content) to area‐based values, a standard in remote sensing and spectroscopic applications (Kattenborn et al. 2019). Photosynthetic pigment contents were expressed in relation to dry needle mass in mg/g (e.g., chl a_M_ for chlorophyll a per dry mass) and in relation to needle area in μg/cm^2^ (e.g., chl a_A_ for chlorophyll a per needle projection area). Water content was calculated as the difference in sample fresh and dry weight, expressed as needle water content (NWC), the mass percentage of water in fresh needle mass, and as EWT in g/cm^2^, i.e., relative to the needle area.

### 2.5 SNP Genotyping Array

The clones under investigation were sampled in 2022 for subsequent genotyping. Approximately 100 mg of needles per sample were cut into small pieces using a scalpel and immediately preserved in liquid nitrogen. These samples were then homogenized for 3 min at 30 Hz using an MM400 mixer mill (Retsch, Haan, Germany). Total genomic DNA was extracted using the NucleoSpin Plant II (Macherey‐Nagel, Düren, Germany) following the manufacturer's protocol.

DNA concentration and purity were quantified employing a NanoDrop 2000 spectrophotometer (Thermo Fisher Scientific, Madison, WI, USA), with a subset of these measurements further validated by Qubit assay (Thermo Fisher Scientific, Madison, WI, USA). The DNA's integrity was assessed on a 0.8% agarose gel. Undiluted aliquots of 45 μL DNA (mean concentration 116 ng/μL, 260/280 ratio between 1.56 and 1.93) were distributed into 96‐well PCR plates.

These plates were then shipped on dry ice to SGS INSTITUT FRESENIUS GmbH, Germany, for analysis. Genotyping was conducted using the PiSy50k SNP chip Axiom array, according to (Kastally et al. 2022). The resulting raw data were provided in CEL file format.

### 2.6 Genomic Relationship Matrix Estimation

We genotyped 74 Scots pine clones using the PiSy50k SNP chip array (Kastally et al. 2022) and applied standard filtering criteria to retain high‐quality SNPs. SNPs with low quality, low minor allele frequency, significant deviations from Hardy–Weinberg equilibrium (*p* < 0.001), and those with heterozygosity exceeding 60% in the populations were removed. After filtering, 20,216 SNPs were retained to construct the GRM ***K*** for the final models, according to Yang et al. (Yang et al. 2010), employing the R‐package AGHmatrix (Amadeu et al. 2016).

The genomic relationship analysis allowed us to discover related clones that originated (most likely) from plus trees standing close to each other in the seed stands. We assumed that all ramets of the exact clone had the same genotype and treated them as such in our analyses. In total, 68 genotyped clones were included in the final analysis.

### 2.7 Statistical Analysis and Genetic Parameters

A linear mixed model (LMM) was fitted to evaluate genotype‐by‐environment interaction in terms of type B genetic correlations (Burdon 1977) implemented in the ASReml‐R package (Butler et al. 2017) as follows:

y=Xβ+Zu+e,

where ***y*** is a vector with the measurements, ***β*** is a vector of fixed effects including overall mean, site, and population within site, and u is a vector of random effects including additive genetic effects nested within site, following $u\sim N\left(0,G\right)$, where ***G*** variance/covariance structure is defined as follows:

$$G=\left[\begin{array}{cc} \sigma_{a_{S_{1}}}^{2} & \sigma_{a_{S_{1}}S_{2}}\\ \sigma_{a_{S_{1}}S_{2}} & \sigma_{a_{S_{2}}}^{2} \end{array}\right]⊗K,$$

where $\sigma_{a_{S_{1}}}^{2}$ and $\sigma_{a_{S_{2}}}^{2}$ are additive genetic variances at site 1 and site 2, $\sigma_{a_{S_{1}}S_{2}}$ is additive genetic covariances between sites 1 and 2, ***K*** is the GRM described earlier, ***e*** is a vector of residuals following $e\sim N\left(0,R\right)$, where ***R*** is the residual variance/covariance structure defined as follows:

$$R=\left[\begin{array}{cc} \sigma_{\xi_{S_{1}}}^{2}\left[\mathrm{AR}1\left(p_{\mathrm{col}}\right)⊗\mathrm{AR}1\left(p_{\mathrm{row}}\right)\right]+\sigma_{\eta_{S_{1}}}^{2}I & 0\\ 0 & \sigma_{\xi_{S_{2}}}^{2}\left[\mathrm{AR}1\left(p_{\mathrm{col}}\right)⊗\mathrm{AR}1\left(p_{\mathrm{row}}\right)\right]+\sigma_{\eta_{S_{2}}}^{2}I \end{array}\right],$$

where $\sigma_{\xi_{S_{1}}}^{2}$ and $\sigma_{\xi_{S_{2}}}^{2}$ are variances for spatially dependent residuals at sites 1 and 2, $\sigma_{\eta_{S_{1}}}^{2}$ and $\sigma_{\eta_{S_{2}}}^{2}$ are variances for independent residuals (nugget) at sites 1 and 2, AR1($p_{\mathrm{col}}$) and AR1($p_{\mathrm{row}}$) are first‐order autoregressive correlation matrices in column and row directions, ***I*** is the identity matrix (Dutkowski et al. 2002), and ***X*** and ***Z*** are incidence matrices associating fixed and random effects.

The genetic correlations between different traits (e.g., type‐A correlation) or between the same trait measured at various ages (e.g., age‐by‐age correlations, in our case limited to the two following years and, thus, further referred to as year‐to‐year correlation) were estimated on pooled data using a bivariate mixed linear model implemented with the ASReml‐R package (Butler et al. 2009) as follows:

y=Xβ+Zu+e,

where ***y*** is a vector with measurements stacked, β is a vector of fixed effects including mean for each trait (or age) and population within trait (or age) nested within trait (or age), u is a vector of additive genetic effects nested within trait (or age) following $u\sim N\left(0,G\right)$, where ***G*** variance/covariance structure is defined as follows:

$$G=\left[\begin{array}{cc} \sigma_{a_{T_{1}}}^{2} & \sigma_{a_{T_{1}}T_{2}}\\ \sigma_{a_{T_{1}}T_{2}} & \sigma_{a_{T_{2}}}^{2} \end{array}\right]⊗K,$$

where $\sigma_{a_{T_{1}}}^{2}$ and $\sigma_{a_{T_{2}}}^{2}$ are additive genetic variances at trait (or age) 1 and trait (or age) 2, $\sigma_{a_{T_{1}}T_{2}}$ is the additive genetic covariances between trait (or age) 1 and trait (or age) 2, ***K*** is the GRM described earlier, and ***e*** is a vector of residuals nested within trait (or age) following $e\sim N\left(0,R\right)$, where ***R*** is the residual variance/covariance structure defined as follows:

$$R=\left[\begin{array}{cc} \sigma_{e_{T_{1}}}^{2} & \sigma_{e_{T_{1}}T_{2}}\\ \sigma_{e_{T_{1}}T_{2}} & \sigma_{e_{T_{2}}}^{2} \end{array}\right]⊗I,$$

where $\sigma_{e_{T_{1}}}^{2}$ and $\sigma_{e_{T_{2}}}^{2}$ are residual variances at trait (or age) 1 and trait (or age) 2, $\sigma_{e_{T_{1}}T_{2}}$ is the residual covariances between trait (or age) 1 and trait (or age) 2, and ***I*** is the identity matrix.

All estimated genetic parameters, including genetic correlations, were tested using a likelihood ratio test (LRT), which compares the final model with a baseline model where the tested parameters are fixed to zero.

Best Linear Unbiased Predictors (BLUPs) of genotypic (clonal) effects were extracted from the fitted LMMs to estimate the performance of individual clones. These BLUPs account for random effects and derive clonal rankings based on genetic merit. Clones are ranked according to their BLUPs, providing a basis for selection decisions (usually in breeding programs; White et al. 2007).

Broad‐sense heritability ($H^{2}$) is calculated using the following equation:

$$H^{2}=\frac{\sigma_{g}^{2}}{\sigma_{p}^{2}},$$

where $\sigma_{g}^{2}$ is the total genetic variation, and $\sigma_{p}^{2}$ is the total phenotypic variation estimated from the model described above. Heritability values range from 0 to 1, where a value of 0 indicates that observed variation is entirely due to environmental or residual effects (i.e., no genetic contribution), and a value of 1 indicates that all phenotypic variation is attributable to genetic differences.

## 3 Results

### 3.1 Genetic Variation in Needle Functional Traits

We estimated the broad‐sense heritability (*H* ^2^) of NFTs utilizing clonal replications in both seed orchards in 2021. For clarity, we divide the traits into two groups: (1) photosynthetic pigment‐related traits (chl a, chl b, car, chl T, car:chl T) in Table 2; and (2) structural and water‐related traits (NL, NWC, LMA, EWT) in Table 3. The tabulated values represent *H* ^2^ of needle traits across different experimental sites. These results indicate the proportion of phenotypic variance in a given trait, which can be attributed to genetic variance among individuals within a population. For instance, when related to dry weight at the Nepomuk orchard (*H* ^2^ Nepomuk), chlorophyll *a*, chlorophyll *b*, and carotenoids showed moderate heritability (0.14, 0.15, and 0.14, respectively). Mass‐based total chlorophyll (chl T_M_) had a heritability of 0.12. Regarding the needle area, *H* ^2^ values are slightly higher in Nepomuk except for chl a_A_. The carotenoid‐to‐total‐chlorophyll ratio (car:chl T) showed the highest heritability of 0.29, suggesting a stronger genetic variation for this ratio. The Plasy orchard (*H* ^2^ Plasy) showed lower or nonsignificant *H* ^2^ values for most studied traits. It is worth noting that car:chl T showed the highest heritability of 0.25 again.

**TABLE 2:** Broad‐sense heritability (*H* ^2^), standard errors (SE), and type B genetic correlations (a measure of G × E) across sites in 2021 of pigment‐related NFTs.

	chl a_M_ [mg/g]	chl b_M_ [mg/g]	chl T_M_ [mg/g]	car_M_ [mg/g]	chl a_A_ [μg/cm^2^]	chl b_A_ [μg/cm^2^]	chl T_A_ [μg/cm^2^]	car_A_ [μg/cm^2^]	car:chl T
*H* ^2^ Plasy (SE)	0.13 (0.09)	NS	NS	NS	0.12 (0.08)	NS	0.10 (0.08)	0.11 (0.08)	0.25 (0.08)
*H* ^2^ Nepomuk (SE)	0.14 (0.07)	0.15 (0.07)	0.12 (0.07)	0.14 (0.07)	0.13 (0.07)	0.22 (0.08)	0.16 (0.08)	0.19 (0.08)	0.29 (0.09)
*H* ^2^ both sites (SE)	0.14 (0.06)	0.13 (0.05)	0.11 (0.05)	0.12 (0.05)	0.13 (0.05)	0.17 (0.06)	0.14 (0.06)	0.16 (0.06)	0.26 (0.07)
Type B gen. corr. (SE)	NS	NS	NS	NS	NS	NS	NS	NS	0.99 (NA)

*Note:* NS = nonsignificant. Chl a—chlorophyll a, chl b—chlorophyll b, chl T—total chlorophyll a + b, car—total carotenoids, car:chl T—ratio of total carotenoids to total chlorophyll. Concentrations of photosynthetic pigments are related to dry weight marked with an M lower index [mg/g] (first four columns on the left) and to needle area projection marked with an A lower index [μg/cm^2^] (following four columns to the right).

**TABLE 3:** Broad‐sense heritability (H^2^), standard errors (SE), and type B genetic correlations across sites in 2021 of structural and water‐related NFTs.

	NL	LMA	NWC	EWT
*H* ^2^ Plasy (SE)	0.35 (0.10)	0.21 (0.09)	0.38 (0.10)	0.15 (0.09)
*H* ^2^ Nepomuk (SE)	0.24 (0.09)	NS	0.15 (0.07)	0.23 (0.09)
*H* ^2^ both sites (SE)	0.29 (0.07)	0.14 (0.05)	0.28 (0.07)	0.20 (0.06)
Type B gen. corr. (SE)	NS	0.99 (NA)	0.72 (0.24)	0.99 (NA)

Abbreviations: EWT, equivalent water thickness; LMA, leaf mass per area; NL, needle length; NS, nonsignificant; NWC, needle water content.

When data from both sites were pooled (*H* ^2^ both sites, Table 2), moderate heritabilities were observed, with slightly higher values for all area‐based traits than mass‐based traits. The highest heritability (0.26) was again estimated for car:chl T. Additionally, a high and significant genetic correlation of type B (0.99) was estimated between both studied sites for car:chl T, implying a robust genetic basis for this specific trait. After combining the sites for analysis, a decreased standard error of *H* ^2^ was noted; the difference was 0.02 on average.

Generally, higher *H* ^2^ was observed in structural and water‐related traits than in the photosynthetic pigment‐related traits. Across both sites, the highest relative value was recorded for needle length *H* ^2^ = 0.29 (SE = 0.07). The highest broad‐sense heritability for a single site was *H* ^2^ = 0.38 (SE = 0.10) for NWC. In contrast to photosynthetic pigment‐related traits, significant type B genetic correlations were estimated for all structural traits except NL. They reached 0.99 for LMA and EWT and 0.72 for NWC, suggesting negligible genotype‐by‐environment interactions and stable expressions of these traits across sites (Table 3).

### 3.2 Spectral Reflectance

#### 3.2.1 Heritability of Needle‐ and Canopy‐Level Reflectance

We estimated *H* ^2^ for each wavelength in the spectral range of 350–2500 nm at the needle level and the spectral range of 429–919 nm at the canopy level.

**FIGURE 2:**
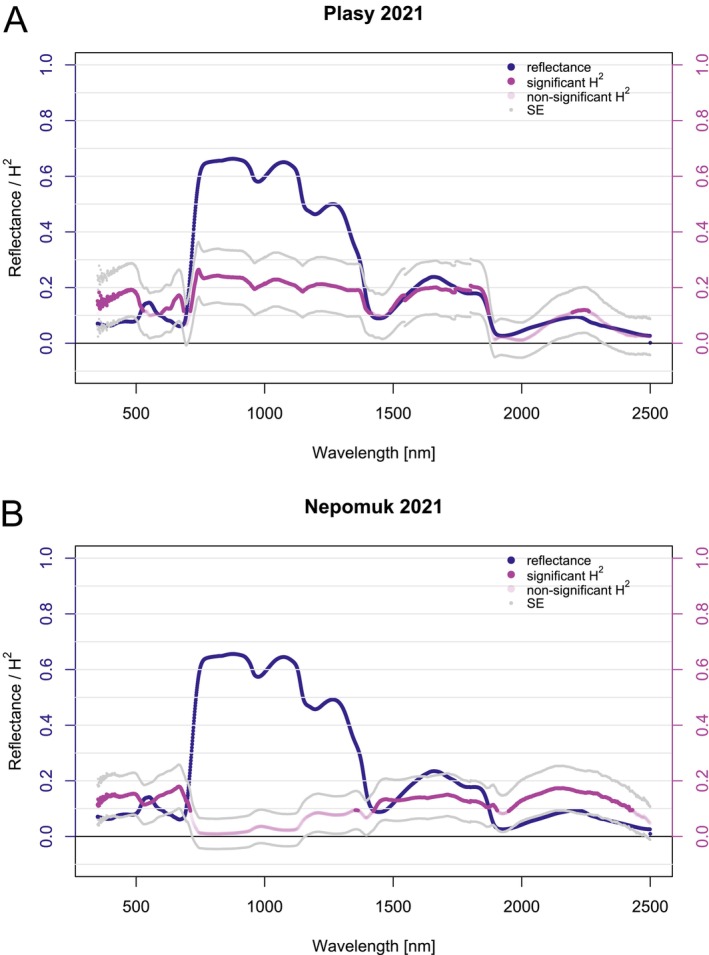
Estimated broad‐sense heritability (*H* ^2^) with standard errors (SE) for needle‐level spectra, shown alongside the mean needle‐level reflectance of all clones at the Plasy (A) and Nepomuk (B) sites in 2021.

In 2021, we analyzed needle‐level reflectance from clones present at both Nepomuk and Plasy orchards. There was a lower sample size at Plasy in 2021, which slightly affected the SE, demonstrated as wider error bars at the Plasy site (Figure 2A) compared to the Nepomuk site (Figure 2B).

At both sites in 2021, the significant heritability of needle‐level reflectance in each wavelength oscillates from 0.10 to 0.25, while Plasy generally exhibited slightly higher values. We observed significant *H* ^2^ estimates across VIS, NIR, and SWIR. However, heritability estimates did not pass the significance threshold (*α* = 0.05) for most NIR wavelengths at the Nepomuk site.

**FIGURE 3:**
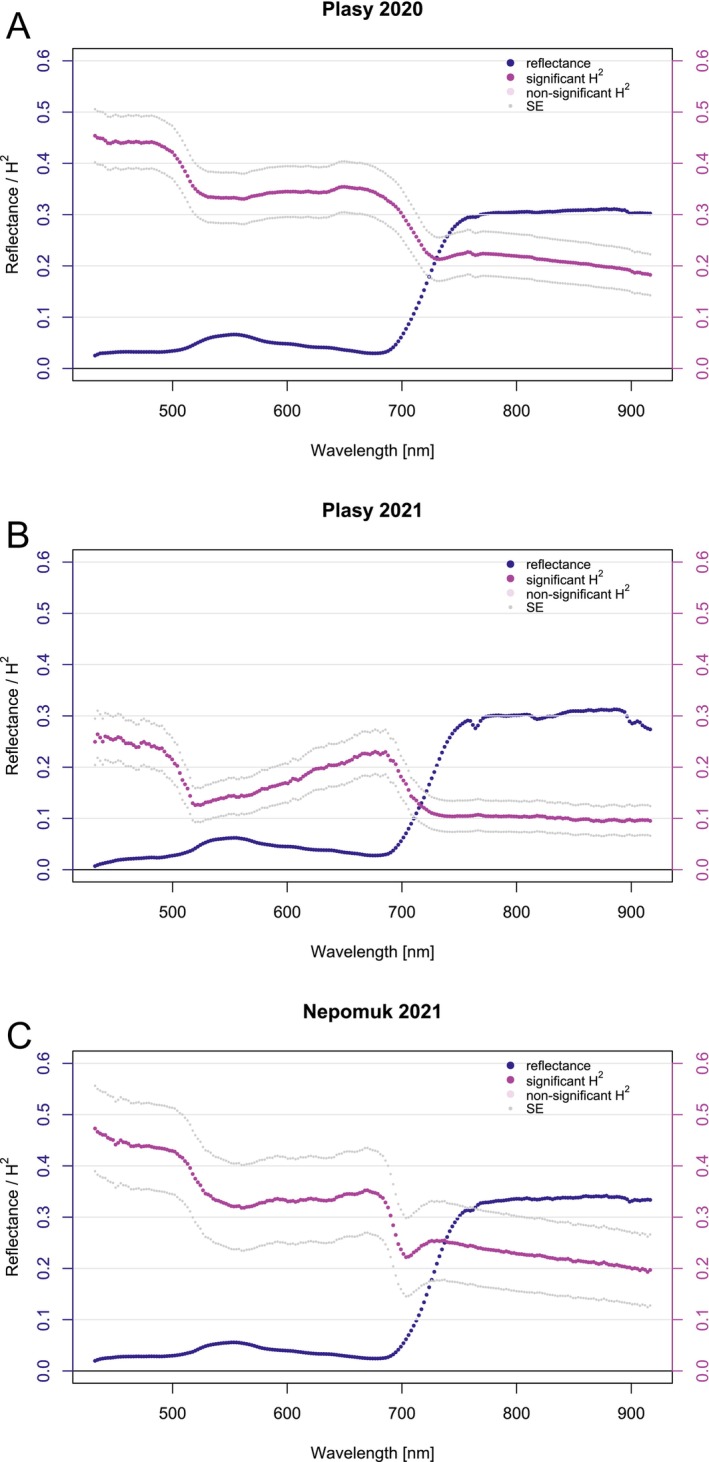
Estimated broad‐sense heritability (*H* ^2^) with standard errors (SE) for canopy‐level spectra, shown alongside the mean canopy‐level reflectance of all clones at Plasy 2020 (A), Plasy 2021 (B), and Nepomuk 2021 (C).

We included all the clones and their replications in the UAV campaign at both sites to assess canopy reflectance heritability. The Nepomuk seed orchard covers a smaller area and contains fewer clones and ramets (Figure S2), resulting in a higher SE for the heritability estimate (Figure 3C). Heritability remained significant across the full spectral range (429–919 nm) at the canopy level (Figure 3). In the VIS region, *H* ^2^ estimates were generally higher from 429 to 500 nm and lower around 520 nm, especially in Plasy 2021. While Plasy 2020 and Nepomuk 2021 showed flatter patterns with higher overall heritability, Plasy 2021 displayed a more distinct trend, resembling that of needle‐level reflectance.

#### 3.2.2 Year‐To‐Year Genetic Correlations of Canopy‐Level Reflectance

Spectral data at the canopy level were collected at Plasy in 2020 and 2021, enabling a detailed analysis of year‐to‐year genetic correlations across the spectral range captured by the camera sensor.

**FIGURE 4:**
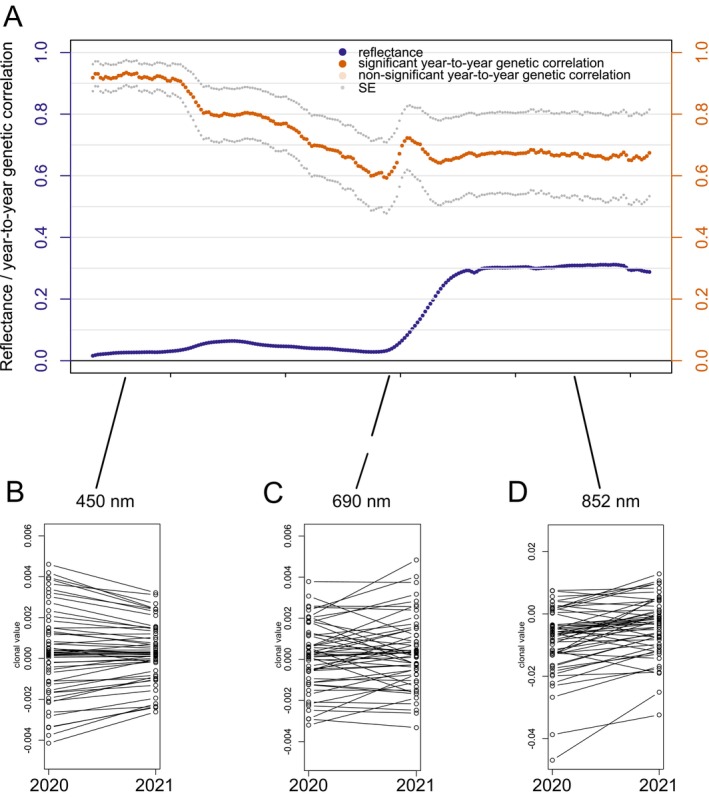
Year‐to‐year genetic correlations with standard errors (SE) estimated at Plasy between 2020 and 2021 are shown in panel (A), alongside the mean canopy‐level reflectance of all clones in these 2 years. Panels (B–D) display the stability of clonal values—representing the predicted genetic component of spectral reflectance—for three representative wavelengths (450, 690, and 852 nm) across the 2 years. These wavelengths (e.g., from VIS, red edge, and NIR regions) were selected to illustrate typical patterns of clonal ranking consistency and variability across the spectral range.

We identified significant positive year‐to‐year genetic correlations primarily in VIS, with correlation values gradually decreasing across longer wavelengths from 0.93 at 450 nm to 0.59 at 690 nm (Figure 4A). At 450 nm (Figure 4B), the rankings of clones based on their BLUP‐estimated genetic values remained consistent across the 2 years, indicating strong genetic stability and aligning with the highest observed year‐to‐year genetic correlation. Conversely, at 690 nm (Figure 4C), the year‐to‐year genetic correlation decreased to a minimum of 0.59, corresponding to more pronounced changes in clonal ranking.

After reaching a minimum in the red‐edge region of the spectrum, the genetic correlation values increased again and formed a local maximum of 0.73. In the near‐infrared (NIR) region, the year‐to‐year genetic correlation remained relatively stable, with an average value of approximately 0.68. Although this indicates moderate consistency in genetic effects across years, a correlation of this magnitude is insufficient to maintain a reliable or stable ranking of clones. (Figure 4D).

#### 3.2.3 Genotype‐By‐Environment Interaction in Needle‐ and Canopy‐Level Reflectance

We estimated type B genetic correlations of hyperspectral reflectance (needle level and canopy level) across the two seed orchards in 2021, focusing on assessing genotype‐by‐environment interaction (G × E).

**FIGURE 5:**
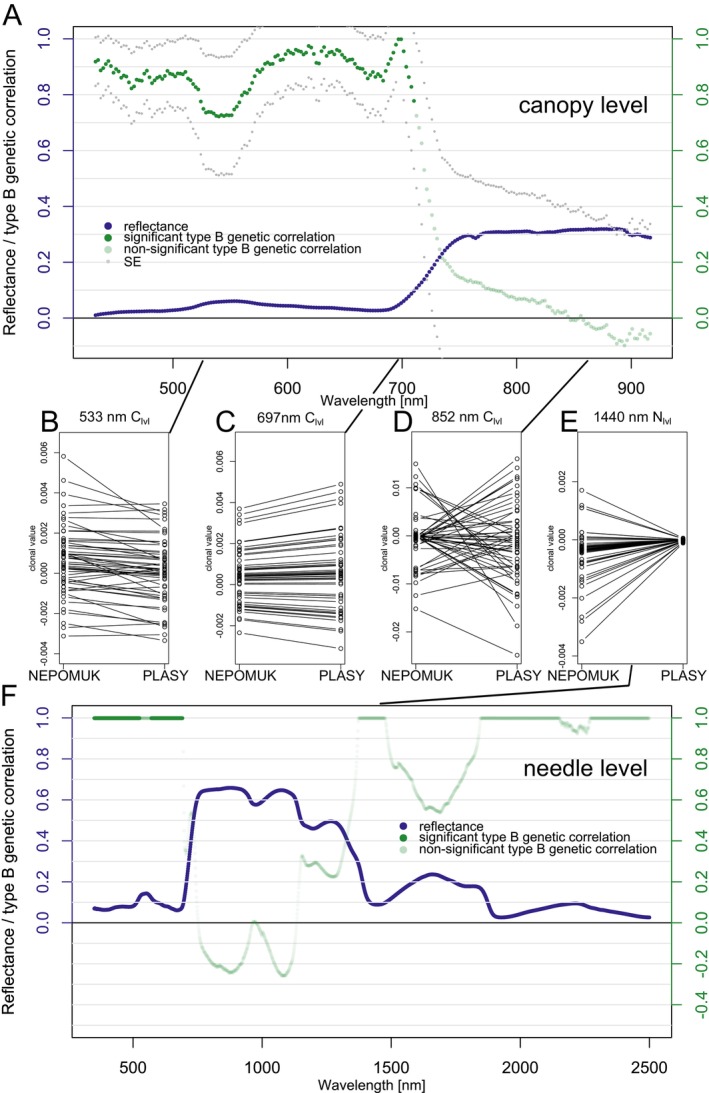
Type B genetic correlation between Plasy 2021 and Nepomuk 2021 as a proxy for GxE interaction in canopy‐level (A) and needle‐level (F) reflectance. The reflectance plotted is the mean of all clones across the sites. Panels (B–D and E) illustrate the stability of clonal ranking for representative wavelengths (533, 697, 852, 1440 nm) across the environments. For needle‐level reflectance, SE was not estimated in VIS, where the type B genetic correlation was significant because the model converged at a boundary. The SE in regions with insignificant type (B) genetic correlations was high and is not visualized.

Significant positive correlations were observed at the canopy level in the VIS range, reaching from 0.72 to 0.99, indicating small to null GxE (Figure 5A). Notably, clonal ranking remained consistent across different environments at wavelengths 533 nm and 697 nm (Figure 5B,C), where type B genetic correlation was high and positive. The strongest type B correlation was observed at 697 nm, reflected in the most stable clonal ranking across sites (Figure 5C). Conversely, in the NIR, type B genetic correlation dropped to zero and negative values, highlighting relevant GxE interactions. This interaction is visible in the clonal value ranking at 852 nm, where some genotypes exhibited high reflectance in one environment but not in another (Figure 5D).

Similar strong, significant (boundary) type B correlations were observed in VIS at the needle level, with a drop to zero and negative values in NIR (Figure 5F). At the needle level, distinct patterns were observed in the SWIR region. In particular, type B genetic correlations reached a value of 1.0 across the major water‐absorption bands centered around 1440, 1900, and 2500 nm. However, these perfect correlations were not statistically significant and should be interpreted cautiously. Clonal rankings at 1440 nm exhibited consistent values but heterogeneous variance between the Nepomuk and Plasy (Figure 5E).

#### 3.2.4 Genetic Correlations Among the Spectra and NFTs

We estimated type‐A genetic correlations between needle‐ and canopy‐level reflectance and photosynthetic pigment content or structural and water‐related needle traits.

**FIGURE 6:**
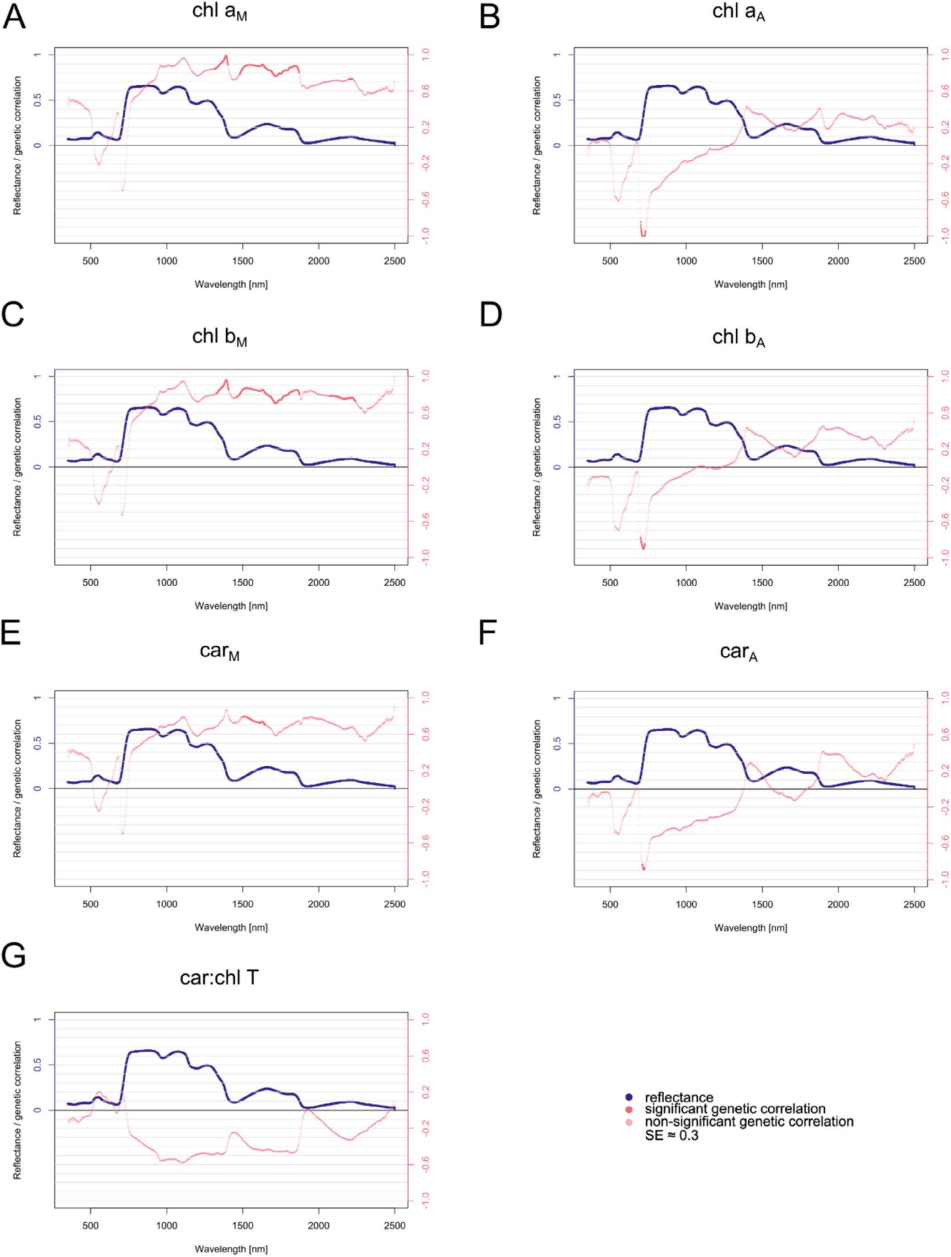
Genetic correlations between needle‐level reflectance spectra and photosynthetic pigments estimated from pooled data (Plasy 2021, Nepomuk 2021) for: chl a_M_ (A), chl a_A_ (B), chl b_M_ (C), chl b_A_ (D), car_M_ (E), car_A_ (F), and car:chl T (G). Concentrations of photosynthetic pigments are related to dry weight marked with an M lower index [mg/g] and to needle area projection marked with an A lower index [μg/cm^2^]. The standard error of genetic correlation reached the average value of 0.3 (not shown). Shades of pink expressed the significance of the genetic correlation. The reflectance plotted is the mean of all clones across the sites.

Needle‐level reflectance correlated significantly with all analyzed photosynthetic pigments (Figure 6). Notably, pigments related to dry matter displayed strong, significant, positive correlations in the SWIR region. In contrast, those related to the needle area exhibited strong, significant, negative correlations in the red‐edge region. Car:chl T did not show a significant genetic correlation across the needle‐level spectral range (350–2500 nm).

**FIGURE 7:**
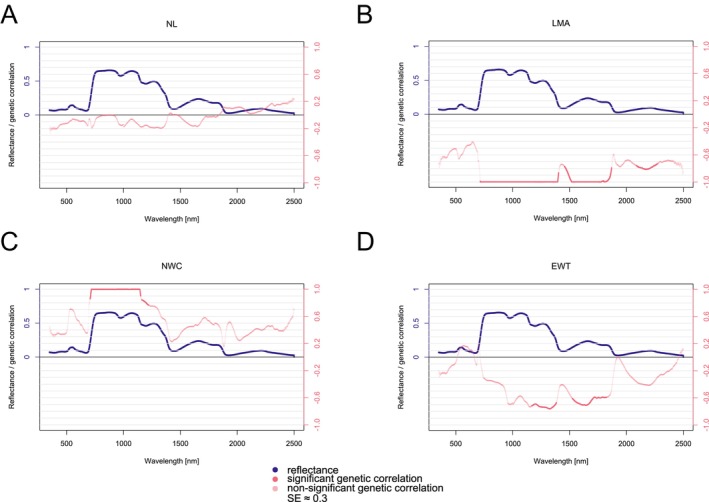
Genetic correlations between needle‐level reflectance spectra and needle structural and water‐related traits estimated from pooled data (Plasy 2021, Nepomuk 2021) for: NL—needle length (A), LMA—leaf mass per area (B), NWC—needle water content (C), and EWT—equivalent water thickness (D). The standard error reached the average value of 0.3 around the genetic correlation value (not shown). Shades of pink expressed the significance of the genetic correlation. The reflectance plotted is the mean of all clones across the sites.

The same analysis was carried out for structural and water‐related traits—LMA showed strong, significant, negative correlations in the NIR and the beginning of the SWIR region at the needle level (Figure 7). Meanwhile, NWC exhibited significant, strong, positive correlations in the NIR. In contrast, EWT showed significant, strong, negative correlations in the later part of the NIR region and the beginning of the SWIR region (Figure 7). NL displayed no significant correlations from the group of structural and water‐related needle traits (Figure 7).

**FIGURE 8:**
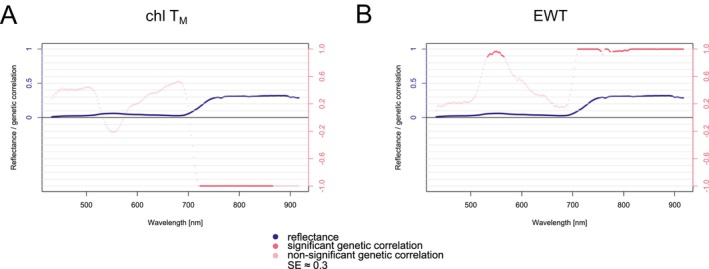
Genetic correlations between canopy‐level reflectance spectra and needle functional traits (NFTs) estimated from pooled data (Plasy 2021, Nepomuk 2021) for: chl T_M_—total chlorophyll a + b (related to dry weight [mg/g]) (A), and EWT—equivalent water thickness (B). The standard error (SE) reached the average value of 0.3 around the genetic correlation value (not shown). Shades of pink expressed the significance of the genetic correlation. The reflectance plotted is the mean of all clones across the sites.

Canopy‐level reflectance only showed significant genetic correlations with chl T_M_ (Figure 8A) and EWT (Figure 8B). Chl T_M_ displayed a negative correlation in the red edge (around 720 nm) and within the adjacent wavebands of the NIR region (the values reached −0.99, and the model reached the boundary; thus, the SEs were not estimated). At the same time, EWT exhibited a positive correlation at the green reflectance peak (around 540 nm) in the VIS spectrum and across most of the NIR region with values of around 0.9 and SEs ranging from 0.23 to 0.30. For comparison, LMA exhibited a similar pattern of genetic correlation as EWT, but the correlation dropped to zero in the NIR region (results not shown). Nevertheless, the correlations did not pass the significance level.

## 4 Discussion

### 4.1 Importance of Genomic Data in the Estimation of Genetic Parameters for Needle Functional Traits Related to Environmental Variation

This study estimated the broad‐sense heritability of various NFTs using clonal replications in seed orchards across two experimental sites in 2021. We incorporated genomic data obtained with the recently developed 50 K SNP genotyping array, allowing for a better understanding of the genetic similarity between clones in the seed orchards. We uncovered a weak relatedness, most likely due to the selection of related plus trees in forest stands originating from natural regeneration, which we accounted for in the models. Given the focus on accurately estimating genomic relatedness, the applied filtering strategy ensured the reliability of SNP data for constructing a robust GRM.

The inclusion of SNP‐based genotyping was motivated by the need to quantify the realized genomic relationships between Scots pine clones, which lack pedigree records due to their historical origin from natural stands. In such populations, traditional pedigree‐based approaches fail to capture hidden relatedness or cryptic structure, potentially biasing estimates of heritability and genetic correlations (De Los Campos et al. 2015; Grattapaglia and Resende 2011). By constructing a GRM from high‐density SNP data, we were able to incorporate this information directly into mixed‐model frameworks to enhance the precision and biological accuracy of quantitative genetic parameter estimation. Moreover, the SNP genotyping framework allowed us to disentangle genetic variance from clonal replicates and contributed to estimating genotype‐by‐environment interactions (G × E) in spectral and needle traits.

### 4.2 Genetic Variation of Photosynthetic Pigment‐Related NFTs

At the Nepomuk site, chlorophyll and carotenoid traits showed low broad‐sense heritability, suggesting modest genetic influence on pigment composition. Sigala et al. (2020) reported variation in pigment pools associated with both provenance and fertilization treatment, corresponding with differences in growth and survival. This suggests that genotypic differences in pigment responses may play a key role in influencing plant fitness under water‐limited conditions.

Photosynthetic pigment composition, specifically the balance of chlorophylls and carotenoids, is closely connected to drought tolerance by supporting the maintenance of photosynthetic function and providing photoprotection in Scots pine (Semerci et al. 2017). Trees experiencing drought stress that maintain higher chlorophyll levels while increasing the carotenoid‐to‐chlorophyll ratio can better prevent chlorophyll breakdown and shield their photosynthetic machinery, which enables sustained carbon assimilation and enhances water‐use efficiency during the acclimation process (Semerci et al. 2017). Among the pigment‐related NFTs in our study, the ratio of carotenoids to chlorophyll (car:chl T) exhibited the strongest genetic signal, suggesting that this integrative metric may capture underlying genetic influences more effectively than individual pigment traits. This aligns with findings from previous conifer studies reporting similar levels of heritabilities for photosynthetic pigments, despite species‐specific physiological differences (Čepl et al. 2018; Hejtmánek et al. 2022). Although cross‐species comparisons should be interpreted with caution, they can reveal general patterns; our findings, alongside others, suggest that pigment traits and their ratios are moderately heritable physiological traits shaped by specific environmental pressures.

At the Plasy site, heritability estimates were generally lower or nonsignificant, reflecting environmental heterogeneity in the larger of the two investigated seed orchards. Another factor could be a smaller sample size, reducing statistical power. Such variation is not unexpected; previous work has emphasized the strong influence of sample size on heritability estimates (Visscher and Goddard 2015). When data from both sites were combined, heritability estimates' precisions improved, demonstrating the value of larger, pooled datasets in detecting genetic signals more reliably. Despite differences between sites, the car:chl T ratio exhibited a very high genetic correlation across environments, indicating minimal genotype‐by‐environment interaction and a stable clonal ranking. This suggests a robust genetic basis for this trait and highlights its potential as a reliable marker of physiological performance under field conditions.

Its ecological relevance is well documented: the car:chl T ratio responds sensitively to environmental stressors such as high light intensity and reflects acclimation strategies during plant development (Demmig‐Adams and Adams 1996; García‐Plazaola et al. 2012). Moreover, the seasonal course of the spectrally detected car:chl T ratio correlated closely with photosynthetic activity in lodgepole pine ( *Pinus contorta* , Dougl.), both in a controlled experiment and field conditions (Gamon et al. 2016). Given its consistent *H* ^2^ across sites and low GxE, the car:chl T ratio is a promising trait for both evolutionary studies and applied breeding. Advances in hyperspectral imaging now enable nondestructive retrieval of this ratio, facilitating large‐scale phenotyping across temporal and spatial gradients (Gitelson 2020; Song and Wang 2022; Sonobe et al. 2020). This positions the car:chl T ratio as a valuable target for monitoring genetic responses to environmental change and for integrating physiological resilience into selection programs.

### 4.3 Genetic Variation of Structural and Water‐Related NFTs

In our study, structural traits generally exhibited higher broad‐sense heritability than pigment‐related traits. Notably, NL demonstrated consistently moderate to high heritability across sites, in line with previous findings in Scots pine (Donnelly et al. 2016). Although exact heritability estimates vary among studies, the general trend supports the notion that needle morphology traits, such as NL, are under considerable genetic influence in conifers, including Scots pine, black pine ( *Pinus nigra* subsp. *pallasiana* (Lamb.) Holmboe), and chir pine (*Pinus roxburghii* Sargent) (e.g., Bhat et al. 2016; Donnelly et al. 2016; Rweyongeza et al. 2003; Yigit et al. 2023).

Given that LMA reflects morphological features, its heritability patterns are likely shaped by similar genetic factors influencing NL, while remaining strongly modulated by the environment. In conifers, LMA primarily reflects needle thickness, tissue density, and degree of lignification (Buraczyk et al. 2022; Houminer et al. 2022). In a provenance common garden study of Scots pine, LMA varied within populations, highlighting its utility for detecting intrapopulation variability (Buraczyk et al. 2022). In our two‐site study, however, significant heritability of LMA was detected only at Plasy in 2021. This site‐ and year‐specific expression of heritable variation suggests that the genetic component of LMA can be masked or amplified by environmental factors, aligning with previous findings that phenotypic variation in LMA reflects genetic differentiation and environmental plasticity. Significant heritability under certain conditions implies that selection could act on LMA in those environments, potentially contributing to adaptation to local climatic constraints. Experimentally induced drought and interspecific comparisons further link high LMA to conservative water‐use strategies and enhanced drought resistance. Indeed, species with larger LMA tend to maintain photosynthetic capacity and midday water potential under water deficit (Bhusal et al. 2021). Hybrids and populations adapted to semiarid environments often exhibit elevated LMA, suggesting a role for LMA in conferring vigor and resilience in marginal sites (Houminer et al. 2022). At the physiological level, persistent xylem impairment can constrain recovery of gas exchange after severe drought, even when water potential partly recovers, highlighting that structural leaf traits (including LMA) interact with hydraulic limitations to determine post‐drought performance (Rehschuh et al. 2020). These lines of evidence support the use of LMA as a practical trait for linking leaf structure, hydraulic function, and drought resilience in Scots pine and other conifers (Bhusal et al. 2021; Buraczyk et al. 2022; Houminer et al. 2022). Additionally, LMA is important for providing information for ecosystem modeling or fire risk management (Féret et al. 2019).

Water‐related traits, while also showing significant genetic control, tended to have lower heritability estimates than structural traits in our dataset. Anatomical adjustments, such as shorter needles and altered mesophyll‐to‐phloem ratios, combined with reduced needle surface area to lower transpiration, enhance water retention under recurrent drought (Bachofen et al. 2021), creating a link between structural and water‐related traits. In our study, NWC exhibited the highest heritability out of the water‐related traits, indicating meaningful genetic variation in traits associated with water retention. However, our values remained moderate, consistent with earlier reports in other pine species such as maritime pine ( *Pinus pinaster* Ait.) (Marguerit et al. 2014). Overall, needle water‐related traits can serve as indicators of drought strategy and recovery potential in pine species (Bachofen et al. 2021; Bhusal et al. 2021), including Scots pine (Rehschuh et al. 2020), and, when heritable, could be leveraged in tree breeding programs aiming for drought resilience.

Our estimated type B genetic correlations further informed the stability of structural and water‐related trait expression across environments, which showed generally high type B values, indicating relatively low GxE interaction. In particular, LMA and EWT demonstrated strong consistency across sites, suggesting stable clonal performance. In contrast, NWC had a slightly lower type B correlation, implying more site‐specific variability. These patterns are consistent with known variation in needle morphology and physiology in Scots pine (Donnelly et al. 2016) and reinforce the potential for selecting stable traits under varying environmental conditions.

### 4.4 Genetic Variation of Needle‐ and Canopy‐Level Reflectance Related to Environmental and Temporal Variation

High‐resolution spectroscopy can capture trait variation at fine scales, potentially revealing underlying genetic differences (Li, Czyż, et al. 2023). By quantifying such variation at fine spatial scales, spectral data can contribute to understanding the evolutionary capacity of populations to respond to environmental change. Previous work in controlled environments has reported high broad‐sense heritability estimates for various multispectral vegetation indices, particularly in apple trees under drought stress (e.g., Coupel‐Ledru et al. 2019; Virlet et al. 2015). However, these values likely reflect the reduced environmental variation in such settings. In contrast, studies on Scots pine and slash pine ( *Pinus elliottii* Engelm.) conducted under more variable field conditions and using half‐sib structures have generally yielded lower, narrow‐sense heritability estimates (Čepl et al. 2018; Tao et al. 2021), suggesting that genetic structure and environmental control play important roles in determining observed patterns of trait heritability.

Our study provides novel insights into the broad‐sense heritability of reflectance in Scots pine, particularly at the canopy level, where estimates reached moderately high values. This represents one of the first assessments of broad‐sense heritability for canopy‐level reflectance in forest trees under natural field conditions. Notably, canopy‐level reflectance consistently exhibited higher heritability than needle‐level reflectance, indicating that trait integration at larger spatial scales may buffer some environmental noise and capture underlying genetic differences more effectively.

Our results revealed spectral patterns, particularly the persistent presence of heritable variation in the red‐edge and visible (VIS) regions, aligning with findings from (Čepl et al. 2018). These spectral regions are closely linked to pigment absorption and stress‐related physiological responses (Čepl et al. 2018; Curran 1989; Curran et al. 1995; Eitel et al. 2011), supporting their utility as targets for genetic and evolutionary studies.

The spatial and temporal consistency of heritability patterns in our dataset further supports the robustness of these spectral features as indicators of genetic variation. For instance, the low heritability observed in the NIR at the Nepomuk site corresponded with the low heritability of NWC, underscoring the link between reflectance traits and underlying physiological processes.

Finally, the significant clonal differentiation in spectral traits observed in our study complements recent evidence of genetic variation in foliar reflectance in other tree species, including interior spruce ( *Picea engelmannii* × *glauca*) (Grubinger et al. 2025) and Fremont cottonwood (Seeley et al. 2024). Together, these findings suggest that reflectance traits, particularly in the VIS and red‐edge regions, are heritable and may also capture adaptive variation relevant to stress tolerance and resource‐use efficiency—traits likely to be under selection in changing environments.

#### 4.4.1 Year‐To‐Year Genetic Correlations

Year‐to‐year genetic correlations provide critical insight into the temporal stability of trait expression, with direct applications in breeding and evolutionary inference. In particular, such correlations help determine whether genetic rankings of individuals or clones remain consistent over time—an essential criterion for selecting genotypes with stable performance across variable environments (Hong et al. 2015). Our study assessed the temporal stability of canopy‐level reflectance by estimating genetic correlations across two consecutive years at a single site. We observed significant, highly positive correlations in both VIS and NIR, with slightly lower values in NIR, indicating that the relative genetic ranking of clones remained largely stable year‐to‐year.

These findings are broadly consistent with previous work demonstrating temporal stability in spectral traits in other species. For example, Song et al. (2024) reported consistent genetic variation in reflectance‐derived phenolic content within a growing season in slash pine. El‐Hendawy et al. (2022) showed stable spectral predictions across years and developmental stages in wheat. Although the temporal resolution and species differ, these studies collectively highlight the potential of spectral traits to capture heritable variation that persists across time and environmental fluctuations.

#### 4.4.2 Gene‐By‐Environment Interactions in Spectral Reflectance

Using type B genetic correlations to quantify G × E, we found that reflectance in the VIS region was highly consistent across our two sites at both canopy‐ and needle‐scales, mirroring the stable VIS performance reported for multispectral indices in slash pine (Tao et al. 2021). This stability implies that pigment‐associated spectral features are under robust genetic control and are therefore promising candidates for operational breeding or physiological performance monitoring.

On the contrary, the type B genetic correlation in the NIR range, in our study, results in lower values, implying substantial genotype‐by‐environment interaction or site‐specific genetic variation in this spectral range on both sites. Changes in needle cellular structure may explain the impact of the environment in the NIR area due to different nutrients, water availability, and overall soil structure at the sites (Buschmann et al. 2012; Granlund et al. 2018). We could explain the nonexistent type B genetic correlation in NIR by insignificant *H* ^2^ in LMA at Nepomuk, as LMA reflects the needle structure. In the next section, we discuss a significant genetic correlation between LMA and needle‐level reflectance in the NIR region, which supports this conclusion. In the SWIR, needle‐level reflectance around major water‐absorption bands (≈1440, 1900, 2500 nm) showed near‐perfect, although insignificant, type B correlations. These results reflect a stable expression of the reflectance of water in the needles and suggest no substantial difference in water availability between the sites. However, this pattern requires validation with a larger sample set.

In contrast to our within‐population investigation, spectral indices at the among‐population level related to photosynthetic efficiency and water content showed significant GxE interactions in a recent study on Fremont cottonwood (Corbin et al. 2024), indicating that different populations and species exhibited distinct strategies when transplanted to new climates.

### 4.5 Genetic Correlation Between NFTs and Spectra

Genetic correlations are often attributed to pleiotropy, where one gene controls the expression of multiple phenotypic traits (Mode and Robinson 1959). The basis for indirect selection in breeding programs lies in significant genetic correlations between traits. In forest trees, studies on genetic correlations between spectral reflectance and physiological traits are scarce.

Our study identified significant genetic correlations between needle‐level reflectance and key NFTs, particularly in the NIR and SWIR regions. These patterns are supported by findings in crop species, where reflectance data have been shown to capture structural and physiological variation in aboveground plant traits reliably. For example, Montes et al. (2022) linked SLA and leaf nitrogen in soybean to hyperspectral signatures, uncovering shared genetic loci that confirmed a pleiotropic basis. An example of forest tree species is a study on slash pine, where genetic correlations were found between growth traits (height and canopy projection area) and various multispectral indices fluctuating in values in different months (Li, Yang, et al. 2023).

Our findings extend this evidence to Scots pine, showing that genetic correlations between LMA and needle‐level reflectance in the NIR and SWIR likely reflect shared genetic control over needle structure and water status. These associations align with patterns seen in Norway spruce, where pigment‐related variation was strongly linked to SWIR reflectance (Hejtmánek et al. 2022), and with studies across multiple broadleaved and coniferous species showing red‐edge sensitivity to pigment content (Zhang et al. 2022). The SWIR region appears to integrate signals from both structural and biochemical components due to overlapping absorption features of water and other leaf constituents.

We observed genetic correlations between reflectance and traits such as EWT and chl T_M_ at the canopy level. To our knowledge, this represents the first report of significant genetic correlations using UAV‐derived canopy spectra in a forest tree context. Previously reported genetic correlations were acquired at the leaf level in trees. Thus, we have not seen a comparable study for a UAV‐based dataset. However, Feng et al. (2017) estimated genetic correlations between image‐based reflectance indices and key agronomic traits, including chlorophyll content in rice.

## 5 Conclusion

This study demonstrates that integrating clonal seed orchards with high‐throughput phenotyping and genomic analyses effectively assesses Scots pine adaptation potential under climate uncertainty. Significant genetic correlations between pigment content, EWT, and canopy‐level reflectance indicate that spectral traits can serve as reliable proxies for indirect selection of adaptive traits. Among NFTs, the carotenoid‐to‐total‐chlorophyll ratio showed the highest broad‐sense heritability, highlighting its genetic basis and potential as a stress‐response indicator, while NWC exhibited even higher broad‐sense heritability, reflecting substantial genetic variation in the drought‐related trait.

Overall, these findings support using spectral data as practical, scalable, and noninvasive tools for assessing adaptive traits. This approach can significantly enhance monitoring evolutionary dynamics and inform selection processes within tree breeding programs. More broadly, the integration of genomic and phenomic data demonstrated here advances our understanding of quantitative trait variation and the mechanisms underpinning local adaptation in long‐lived tree species confronted with rapid environmental changes.

## Conflicts of Interest

The authors declare no conflicts of interest.

## Supporting information


**Table S1:** Soil analysis results from Plasy and Nepomuk seed orchards from 1988 and 2015. The Forestry and Game Management Research Institute prepared data from 1988. For the 2015 data, *n* = 6 (Nepomuk), *n* = 7 (Plasy). The ‐ symbol indicates that the analysis was not performed.
**Figure S1:** Spatial visualization of the Plasy seed orchard design and the clones utilized for the analyses.
**Figure S2:** Spatial visualization of the Nepomuk seed orchard design and the clones utilized for the analyses.

## Data Availability

The data supporting the findings of this study are openly available in Figshare at https://doi.org/10.6084/m9.figshare.27134907.v2.
