# Supplementary material for: Needle‐ and Canopy‐Level Genetic Variation in Scots Pine (Pinus sylvestris L.) Revealed by Hyperspectral Phenotyping Across Sites and Seasons
Source: Evol Appl. 2025 Nov 12;18(11):e70176. doi: 10.1111/eva.70176 (PMC12612600; doi:10.1111/eva.70176)
Supplement: Supplementary file 1 — Table S1: Soil analysis results from Plasy and Nepomuk seed orchards from 1988 and 2015. The Forestry and Game Management Research Institute prepared data from 1988. For the 2015 data, n = 6 (Nepomuk), n = 7 (Plasy). The ‐ symbol indicates that the analysis was not performed. Figure S1: Spatial visualization of the Plasy seed orchard design and the clones utilized for the analyses. Figure S2: Spatial visualization of the Nepomuk seed orchard design and the clones utilized for the analyses. [file EVA-18-e70176-s001.docx]

Supplementary Materials

*Suppl. Table 1: Soil Analysis Results from Plasy and Nepomuk seed orchards from 1988 and 2015. The Forestry and Game Management Research Institute prepared data from 1988. For the 2015 data, n=6 (Nepomuk), n=7 (Plasy). The - symbol indicates that the analysis was not performed.*

|  | 1988 | | 2015 | |
| --- | --- | --- | --- | --- |
|  | Plasy | Nepomuk | Plasy | Nepomuk |
| exchange acidity of soil - pH | 3.3 | 3.6 | 3.7 | 3.8 |
| Calcium content (mg Ca/kg) | 263 | 293 | 441 | 660 |
| Magnesium content (mg Mg/kg) | 50 | 41 | 53 | 121 |
| Phosphorous content (mg P/kg) | 11 | 36 | 19 | 9 |
| Potassium content (mg K/kg) | 20 | 43 | 83 | 92 |
| Carbon content (% C) | - | - | 1.7 | 1.3 |
| Nitrogen content (% N) | 0.05 | 0.08 | 0.11 | 0.16 |
| Hummus content (%) | 1.7 | 1.61 | 3 | 2.2 |
| C:N ratio | 20 | 8 | 14 | 8 |

**
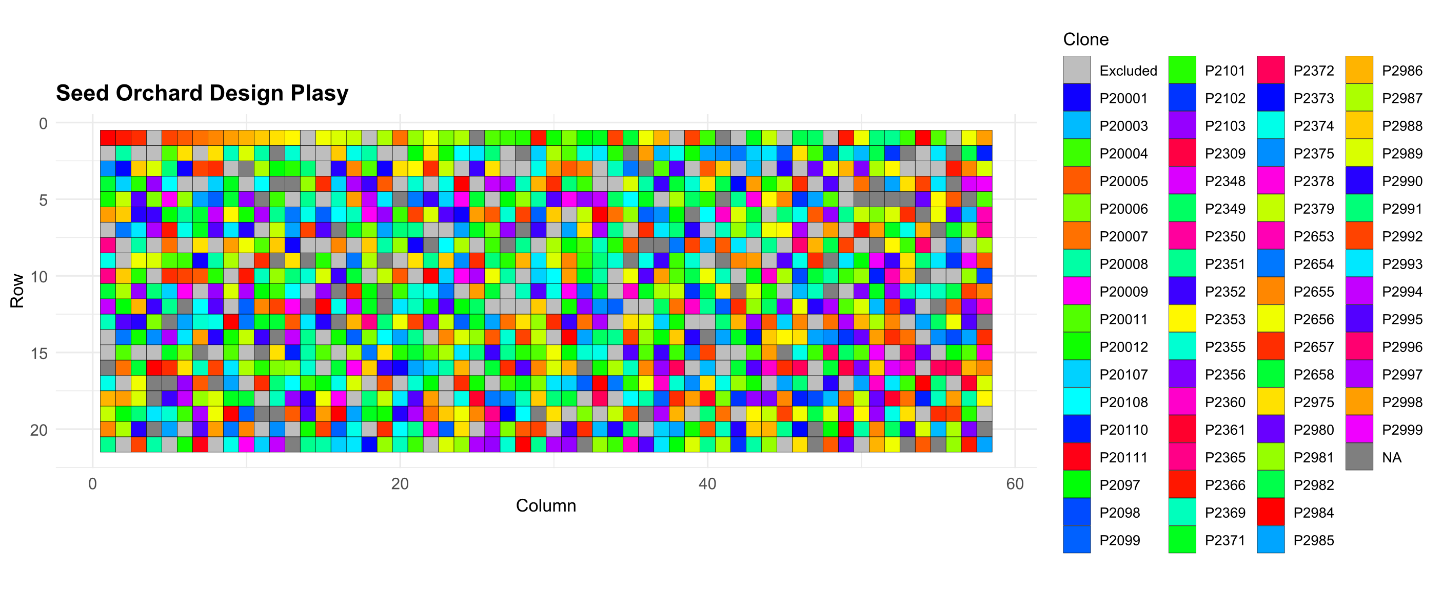
**

Suppl. Fig. 1: Spatial visualization of the Plasy seed orchard design and the clones utilized for the analyses.

**
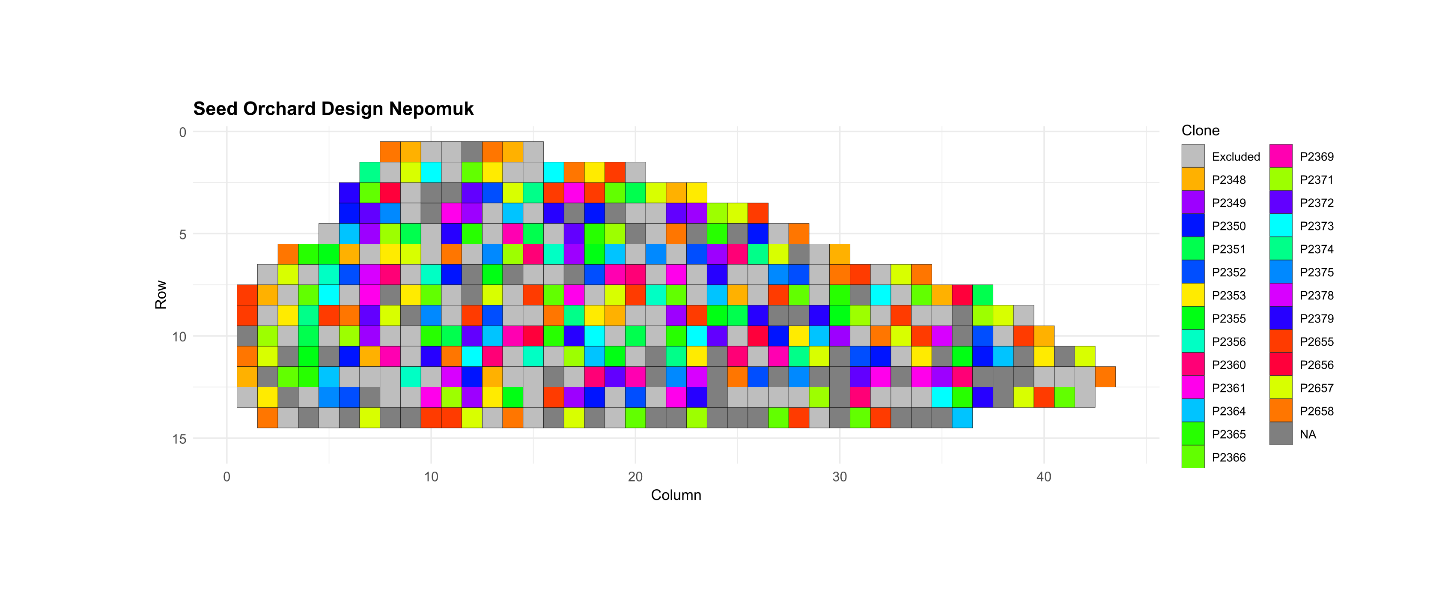
**

Suppl. Fig. 2: Spatial visualization of the Nepomuk seed orchard design and the clones utilized for the analyses.
